# Prevalence of Frailty among Community-Dwelling Older Adults in Asian Countries: A Systematic Review and Meta-Analysis

**DOI:** 10.3390/healthcare10050895

**Published:** 2022-05-12

**Authors:** Thi-Lien To, Thanh-Nhan Doan, Wen-Chao Ho, Wen-Chun Liao

**Affiliations:** 1 College of Public Health, China Medical University, Taichung 406040, Taiwan; u108050101@cmu.edu.tw (T.-L.T.); drchannhan@gmail.com (T.-N.D.); wcho@mail.cmu.edu.tw (W.-C.H.); 2 Faculty of Nursing, Duy Tan University, Danang 550000, Vietnam; 3 Department of Rehabilitation, Quang Nam Northern Mountainous Region General Hospital, Quang Nam 560000, Vietnam; 4 School of Nursing, China Medical University, Taichung 406040, Taiwan; 5 Department of Nursing, China Medical University Hospital, Taichung 404332, Taiwan

**Keywords:** frailty, prevalence, older adults, community dwelling, systematic review, meta-analysis

## Abstract

This study aimed to synthesize frailty prevalence among community-dwelling older adults in Asia and identify factors influencing prevalence estimates. Five electronic databases were searched by 29 April 2022, including representative samples of community-dwelling adults who were aged 60 years and older and lived in Asia. Cross-sectional or national longitudinal population-based cohort studies completed with validated instruments were selected. Twenty-one studies with 52,283 participants were included. The pooled prevalence rate of frailty was 20.5% (95% CI = 15.5% to 26.0%). The estimated frailty prevalence was 14.6% (95% CI = 10.9% to 18.8%) while assessed by the Fried frailty phenotype, 28.0% (95% CI = 21.3% to 35.3%) by the Cumulative Frailty Index, 36.4% (95% CI = 33.6% to 39.3%) by the Study of Osteoporotic Fractures (SOF) index, and 46.3% (95% CI = 40.1% to 52.4%) by the Clinical Frailty Scale (*p* < 0.01). Subgroup analysis in studies using the Fried’s phenotype tool found that frailty prevalence was increased with older age (*p* = 0.01) and was higher in those who were single (21.5%) than in married participants (9.0%) (*p* = 0.02). The study results supported a better understanding of frailty prevalence in different geographical distributions and provide references for health policy decision-making regarding preventing frailty progression in older adults.

## 1. Introduction

Frailty is an age-related health issue characterized by deteriorating reserve and resiliency capacities and an increased vulnerability to stressors, highly prevalent in older adults [1,2]. Over the past decade, frailty has substantially attracted research interest because it increases the risks of various adverse health outcomes, including falls, hospitalization, and mortality [1,3,4]. 

There has been no universal consensus on standardized screening and assessment tools for frailty in the elderly [5]. However, there are two popular instruments for the operational definitions of frailty [6]. The Fried frailty phenotype is the popular single-dimensional approach, which is oriented mainly to the physical attributes and capabilities characterized by the following five phenotypic criteria: weight loss, slowness, weakness, physical inactivity, and exhaustion; wherein frailty is defined as the presence of at least three criteria [1]. A Cumulative Frailty Index, including symptoms, disease, disability, and signs, is calculated by dividing the sum of the health deficit scores by the total number of deficits [7].

Many recent studies have identified factors associated with frailty, including sociodemographic, clinical, lifestyle, psychological, and biological factors [8]. Advancing age, being female, lower education level, and living alone were associated with an increased prevalence of frailty [9,10]. An unhealthy lifestyle characterized by dietary problems, smoking, and alcohol use, can lead to the onset of frailty [9,11]. Investigations of factors correlated to the development of frailty are needed to determine high-risk groups.

Studies related to the prevalence of frailty have enormously grown globally. Regional statistics have reported that the prevalence of overall frailty was 19.6% in community-dwelling older adults aged 60 years and above in Latin America and the Caribbean (LAC) [12], but 12% in the European community population aged 50 or above [13]. Variation and heterogeneity in frailty measures and participant characteristics exist among studies. A recent systematic review reported a pooled prevalence of 12% by using physical frailty measures and 24% by using the deficit accumulation model among individuals aged ≥ 50 years in 62 countries across the world [14]. In this report, the estimated prevalence of frailty among those aged ≥ 50 years in Asia was 11% using the physical frailty model and 25% using the deficit accumulation model, which was higher than in Europe which was 8% and 22%, respectively [14]. Frailty assessment tools used in different studies, including the Fried Phenotype [1], the Osteoporotic Fractures (SOF) index [15], the Clinical Frailty Scale [16], and the Cumulative Frailty Index [7], affect the prevalence of frailty but may be the best fit to the associated countries or regions. Lifestyles and cultural differences among different regions may also be factors to affect the prevalence of frailty. A study regarding Asians who share similar characteristics may provide references for future policymaking.

Asia is the most populous region in worldwide, with 60% of the world’s population in 2011 and 55% in 2050 [17]. It is expected to double its population that is aged 60 or over from 549 million to nearly 1.3 billion between 2017 to 2050 [18]. Older adults within 60 to 80 years are a growing group and can become society’s resources and not only burdens. Prevalence of frailty status affects national productivity and increases society’s burdens. Most meta-analysis studies merged all available studies together, including regional and national data, to estimate frailty prevalence. It may over- or underestimate the prevalence of frailty. A representative population-based study was suggested [14]. This systematic review and meta-analysis aimed to provide an updated view of the overall prevalence of frailty among older adults aged 60 to 80 years in Asian communities by merging representative population-based data, and subgroup estimates based on demographic characteristics, lifestyles, geographical distribution, and frailty assessment methods. There were two research questions to address:

1. What is the overall prevalence of frailty among community-dwelling adults living in Asian countries?
2. What factors are associated with the overall prevalence of frailty among community-dwelling adults in Asian countries?

## 2. Materials and Methods

### 2.1. Register and Protocol

This review protocol was registered at PROSPERO (International Prospective Register of Systematic Reviews) with registration number CRD42020176803. This systematic review and meta-analysis followed the PRISMA (Preferred Reporting Items for Systematic Reviews and Meta-Analyses) [19] and Meta-analysis of Observational Studies in Epidemiology (MOOSE) reporting guidelines [20].

### 2.2. Data Sources and Search Strategy

A systematic literature search in the following five electronic databases was conducted: PubMed, Embase, Cochrane Library, CINAHL, Ovid Medline, with restriction to the English language between 1 January 2010, and 31 December 2020, involving humans only. The last update of searching was 29 April 2022. Reference lists of all included papers and critical systematic review papers were hand-screened to retrieve relevant studies. We also emailed corresponding authors to request any missing data, as necessary. Ethical approval was not required because study participants are based on published studies. The search strategy was adapted for each database and first developed in PubMed with the following search terms: ((((Prevalence) OR (Incidence)) OR (epidemiology))) AND ((adult, frail older) OR (elderly, frail))). The strategy was modified according to the individual requirements of different databases (Table S1). 

### 2.3. Eligibility Criteria

In the first selection round, we considered studies eligible if they met the following general criteria (1) focused on community-dwelling older adults; (2) recruited individuals in Asia; (3) reported frailty prevalence as the primary outcome of the study. Studies were ineligible if (1) their participants were hospitalized, institutionalized, or nursing home residents; (2) older adults with specific diseases or conditions; (3) editorials, commentaries, review papers, correspondence, abstract-only publications, conference proceedings, conference abstracts, personal opinions, randomized controlled trial studies were also excluded. In the second selection round, studies were selected to satisfy all the following inclusion criteria: (1) the study sample were older persons defined as ≥60 years of age according to United Nations [21]. We also included studies that enrolled the younger populations in which they separately reported the prevalence of frailty in adults aged 60 years or above. We minimize collecting the octogenarians and nonagenarians because these populations are a much higher prevalence of frailty; (2) studies were cross-sectional studies or community-based longitudinal studies with a baseline assessment of the prevalence of frailty from nationally representative samples of participants older than 60 years. Multiple studies of the same cohort, which obtained more detailed information (about the participants and the used frailty definition) and more complete data on the largest sample size, were selected. We collected the data from a sample representing the whole population as a national survey; otherwise, regional studies were extracted. (3) In cases where a study assessed the prevalence of frailty by two or more different scales, we leaned towards the prevalence assessed by physical frailty. (4) Frailty was defined clearly by any validated instrument. Individuals must be classified into three categories of frailty: non-frail, prefrail, and frail.

### 2.4. Study Selection Methods

The study selection was carried out in two independent reviews (W.-C.L. & T.-L.T.). After removing duplicate records, two reviewers independently screened all the titles and abstracts based on the general eligibility criteria in the first selection round. We selected potentially relevant papers for full-text reading, which were then independently assessed for eligibility by two authors (W.-C.L. and T.-L.T.) in the second round using the aforementioned inclusion criteria. Any disagreements were resolved through discussion.

### 2.5. Methodological Quality Assessment

The quality of each study was assessed by one author (T.-L.T.) and then verified by a second author (W.-C.L) using The Joanna Briggs Institute’s Critical Appraisal Checklist for the prevalence studies [22]. The reviewers scored 1 for “Yes” and 0 for “No” or “Unclear” or “Not applicable”. This checklist addresses critical issues, including representative sample, participant recruitment, sample size, study subjects and setting, data coverage of the identified sample, measurement of the condition, statistical analysis, and the identification of confounding factors/subgroups/differences. A study was considered “adequate quality” if it met more than five out of nine items and became a potential study for this systematic review.

### 2.6. Data Extraction

Data were extracted from each paper by the investigator (T.-L.T.) onto formatted spreadsheets in Excel files, including the first author and year of publication, country, United Nations subdivisions, adequate sample size, age (mean or median and range), percentage of women, frailty assessment methods, the prevalence data, prevalence of frailty stratified by age groups and gender if available, and study quality assessment score. The second author (W.-C.L.) subsequently checked for completeness again. Any disagreements were discussed until reaching a consensus.

### 2.7. Data Synthesis and Analysis

A random-effects model using the DerSimonian–Laird method estimates with 95% CI was chosen to calculate the pooled prevalence of frailty because heterogeneity was anticipated [23]. Statistical analysis was performed using the meta-package [24] in RStudio version 1.3.1093 (Integrated Development for R. RStudio, PBC, Boston, MA, USA) and the Freeman–Tukey double arcsine transformed to stabilize variance [25]. To measure heterogeneity among studies, we used Cochran’s Q-statistic and the Chi-square test, a p-value less than 0.05 was statistically significant [26]. I-squared (I^2^) was to quantify the magnitude of inconsistency in effect sizes, in which 25%, 50%, and 75% were considered low, moderate, and high degrees of heterogeneity, respectively [26]. We used the Funnel plot and Egger’s weighted regression test to explore the possibility of publication bias [27]. The result of Egger’s test indicates that symmetry exists in the funnel plot with *p* > 0.05. Outliers have the potential to be an actual influence via the set of leave-one-out diagnostic tests. 

Chi-squared tests were used to test for differences between subgroups, and a *p*-value less than 0.05 indicates it is statistically significant [28]. Subgroup analyses on the prevalence of frailty were carried out to discover possible heterogeneity sources and interpret the variability among studies in the systematic review. Subgroups were classified according to age (60–64 versus 65–69 versus 70–74 versus 75–79 versus 80–84 versus 85+ years old), gender (male versus female), marital status (married versus single), living arrangement (living alone versus living with family), smoking status (no smoking versus current smoking), alcohol drinking (no drinking versus current drinking), study regions (Eastern Asia versus Southern Asia versus South-Eastern Asia versus Western Asia), and frailty assessment methods (Fried frailty phenotype (original and modified) versus Cumulative Frailty Index versus Study of Osteoporotic Fractures (SOF) index versus Clinical Frailty Scale). According to the United Nations, the Asian region is shared by 48 countries divided into six main geographical sub-regions: Northern Asia, Central Asia, Eastern Asia, South-Eastern Asia, Southern Asia, and Western Asia [29].

## 3. Results

### 3.1. Selection Process

**Figure 1:**
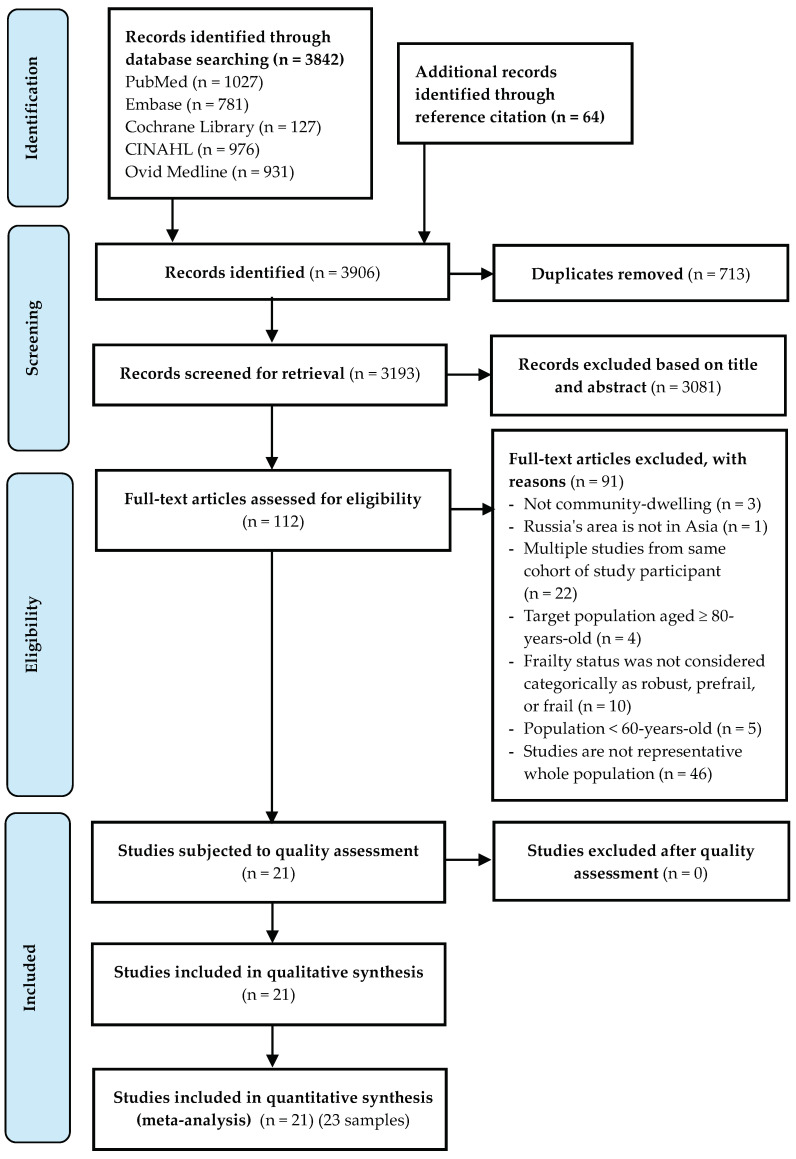
PRISMA flow diagram of the Study Selection Process.

The literature search has yielded 3842 records through electronic databases and 64 records in citation reference. After removing duplicated and screening titles and abstracts, 112 full-text articles were assessed for eligibility as described in a PRISMA flow diagram (Figure 1); 21 studies met the eligibility. All included studies were classified as high to moderate quality, with the number of “yes” answers ranging between 5 and 9. The methodology quality of studies was provided in more detail in Table S2. One study reported three separate samples [30]. Therefore, twenty-one studies with twenty-three samples were included in the systematic review.

### 3.2. Study Characteristics

Table 1 presents the characteristics of 21 studies with 23 representative samples included in this systematic review. There were a total of 52,283 older adults with the median sample size across studies being 1120 (range 250–11,165). Twenty-one samples reported the mean (median) age of the study population, ranging from 66 to 75.7 years. The proportions of women were 53.2% involving 27,829 participants. All studies used a nationally and regionally representative elderly population. Figure S1 shows the geographical distribution of the population of included papers. Seven samples were conducted in Eastern Asia [30,31,32,33,34], eight samples in South-Eastern Asia [35,36,37,38,39,40,41,42], five samples in Southern Asia [43,44,45,46,47], and three samples in Western Asia [48,49,50]. Twelve samples of this review used the Fried frailty phenotype to define frailty [31,33,35,36,37,38,39,41,42,44,46,50]; seven samples used the Cumulative Frailty Index [30,32,34,40,47]; one sample used both the Fried frailty phenotype (four criteria, except shrinking) and FRAIL scale [49]; one sample used all three scales: the Fried frailty phenotype, Cumulative Frailty Index, and Tilburg Frailty Indicator [43]; whereas two samples used other scales [45,48]. Criteria in studies that used the Cumulative Frailty Index varied between 30 and 44 deficits. Data were stratified by age in 18 samples and gender in 22 samples. We emailed 11 corresponding authors to enquire about data that was not in the manuscripts, and we received responses from six authors. Important data, such as the prevalence of frailty stratified by age [44,48] and the mean age of the target population [36] were obtained from the responses. More details of these studies were included in the meta-analysis shown in Table S3.

### 3.3. Prevalence of Frailty

**Figure 2:**
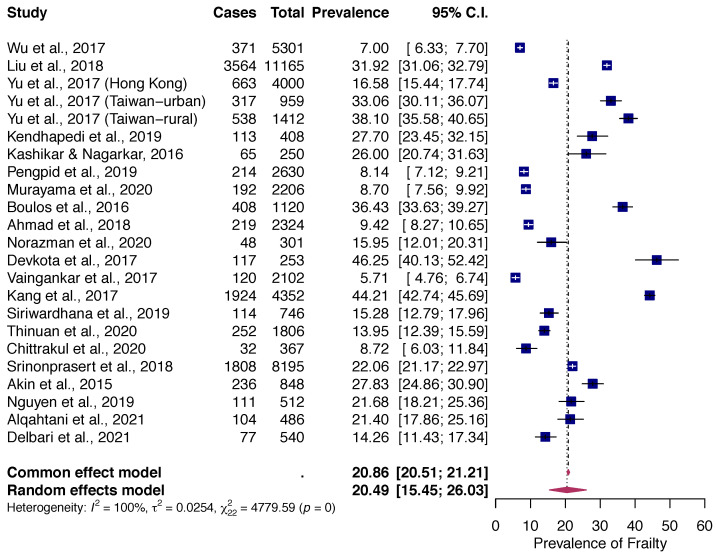
Forest Plot of Overall Pooled Prevalence of Frailty [30,31,32,33,34,35,36,37,38,39,40,41,42,43,44,45,46,47,48,49,50].

**Figure 3:**
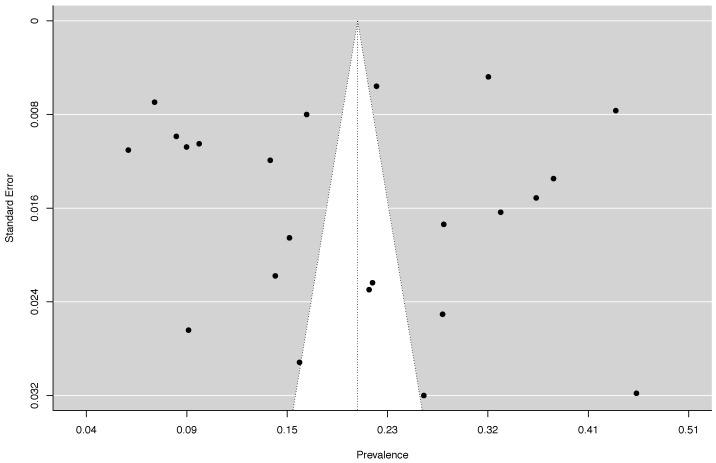
Funnel Plot of Prevalence Rates of Frailty Among Community-Dwelling Older Adults in Asian countries. P for Egger’s test = 0.63.

The random-effects pooled prevalence of frailty in Asian was 20.5% (95% CI: 15.5% to 26.0%, Q = 4779.59, df = 22, *p* < 0.000001; I^2^ = 99.54%) with a range from 5.7% (Singapore) to 46.2% (Nepal) in studies reviewed (Figure 2). There was no evidence for the existence of publication bias (Figure 3), or any single study substantially influencing the overall prevalence of frailty (Table S4).

### 3.4. Factors Associated with Prevalence of Frailty

#### 3.4.1. Frailty Assessment Methods

**Figure 4:**
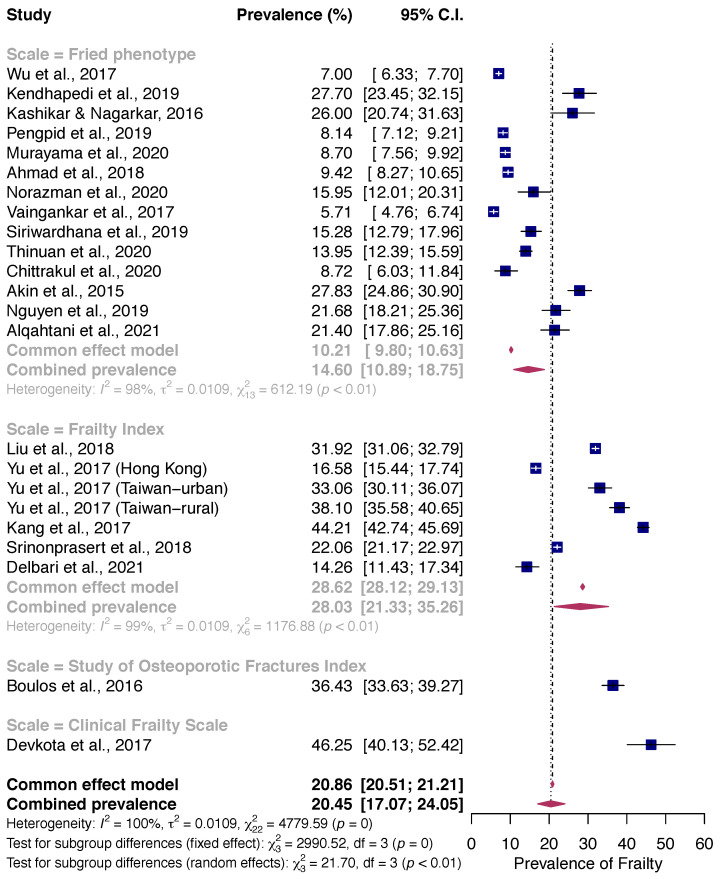
Pooled Prevalence of Frailty stratified by Frailty Assessment Method among community-dwelling older adults [30,31,32,33,34,35,36,37,38,39,40,41,42,43,44,45,46,47,48,49,50].

Subgroup analysis based on frailty assessment methods revealed that frailty prevalence assessed by the Fried frailty phenotype (14.6%) was lower than that assessed by the Cumulative Frailty Index (28.0%). Two studies using the Study of Osteoporotic Fracture (SOF) index and Clinical Frailty Scale also reported a higher frailty prevalence (36.4% and 46.3%) (*p*-value for difference < 0.01) (Figure 4).

#### 3.4.2. Geographic and Socio-Demographic Characteristics and Lifestyle Factor

The majority of the included studies reported the frailty prevalence using the Fried frailty phenotype (n = 14), employing narrower confidence intervals. Therefore, our review stratified across these 14 studies via subgroups by geographical regions, social-demographic characteristics (age groups, gender, marital status, and living arrangement) and lifestyle factors (smoking status and alcohol drinking) to better investigate potential sources of heterogeneity.

Pooling prevalence of frailty using the Fried frailty phenotype was stratified (Table 2). Subgroup analysis revealed high heterogeneity in all analyzed categories. The frailty prevalence increased with age from 8.1% to 34.0% by 5-year age ranges, accordingly (with *p* for difference = 0.01). A higher prevalence was observed in older adults who were single (21.5%) than were married (9.0%) (with *p* for difference = 0.02). No difference was found in the prevalence of frailty when stratified by gender, living arrangements, smoking status, and alcohol drinking (with *p* for difference > 0.05).

Further analysis by stratified geographical regions of Asia showed that frailty prevalence among community-dwelling elderly populations was 22.5% in Southern Asia (India [43,44], Sri Lanka [46]), 24.6% in Western Asia (Saudi Arabia [50], Turkey [49]), 11.3% in South−Eastern Asia (Indonesia [35], Malaysia [36,37], Singapore [42], Thailand [38,39], Vietnam [41]), and 7.8% in Eastern Asia (China [31], Japan [33]).

## 4. Discussion

This study assessed the frailty prevalence in community-dwelling older adults aged 60 years and older in Asian countries and identified factors that influence prevalence estimates among countries. The average prevalence of frailty was 20.5% (95% CI = 15.5% to 26.0%). The prevalence of frailty was lower when using the Fried phenotype (14.6%) and increased with age and in those who were single. Our findings indicate that approximately one in every five community-dwelling older adults in Asian areas may have frailty, which is roughly equal to those reported in Latin America and the Caribbean (LAC) (19.6%; 95% CI = 15.4% to 24.3%; range: 7.7% to 42.6%) [12]. However, the prevalence yielded in our study is relatively higher than that estimated in Europe, North America, and Oceania (10.7%; 95% CI = 10.5% to 10.9%) [10], 22 European countries (12%; 95% CI = 10% to 15%) [13], 62 countries and territories worldwide (17%; 95% CI = 16% to 18%) [14] and low and middle-income countries (the majority from Brazil) (17.4%; 95% CI = 14.4% to 20.7%; range: 3.9% to 51.4%) [51]; but lower than those reported in non-institutionalized Brazilian adults aged 60 and older (24%; 95% CI = 21% to 26%) [52]. 

A wide range of frailty prevalence was found among the included studies, which could be attributable to the high heterogeneity of the samples included in this review, thus further subgroup analyses by risk factors associated with frailty are performed in this study. The frailty tools, geography regions, and population characteristics could potentially impact the reported prevalence of frailty.

The frailty assessment method was found to significantly affect the prevalence of frailty, which is consistent with previous research [6,10,52]. In comparison to other methods, evaluation using the Fried phenotype indicated a lower prevalence (14.6%). Two reviews of global frailty prevalence reported that using the physical phenotype generated a lower estimated prevalence (9.9% and 12%) than the broad phenotype of frailty (13.6% and 24%) [10,14]. With a long checklist of clinical diseases and illnesses, the Cumulative Frailty Index is thought to be more sensitive than a physical phenotype based on a predefined set of five criteria. This might explain why the Cumulative Frailty Index produces higher estimates [6]. Regarding the use of the deficit accumulation approach, the variation in the number of deficits and cut-off points for defining frailty status was inconsistent among studies. 

In this meta-analysis, the prevalence of frailty in those aged 80–84 years and ≥85 years was 29.8% and 34.0%, respectively, which roughly doubled the rate in individuals aged 70–74 years and 75–79 years with a prevalence of 14.4% and 19.2%, respectively. Frailty was shown to be more prevalent as people became older and substantially increased after the age of 75 years, which is also reported in previous reviews [10,14,51,53,54,55]. This is most likely due to the fact that as people age, they gradually undergo age-related degenerations, and their functional residual capacities ceaselessly decline [53]. Age 80 seems to become a cut point for frailty. After the eighties, people accumulate deficits and become more vulnerable to adverse health outcomes [56]. Asian nations, in general, and Japan, are expected to have a high life expectancy, which results in many frail older people surviving into their 80s or 90s, leading to a higher prevalence in these groups [54]. Literature suggested that frailty, but not chronological age, is a prognostic risk factor for complications after elective surgery [57,58]. Future research should develop interventions targeting age groups of the eighties to prevent or reduce frailty.

Our results also found that frailty was associated with marital status, with a higher prevalence in older adults who were single than in married people, which is aligned with a previous study [35]. We may emphasize the beneficial impacts of structural support from social interactions as one of the probable explanations for the lower frequency of frailty and the presence of married relationships [59]. Inversely, the gender-stratified weighted prevalence of frailty showed no significant difference between men and women, which is in line with the previous reviews [12,52]. Women’s life expectancy is higher than men’s, even though women also suffer from a variety of chronic conditions in their later life [60]. Thus, gender-related differences in frailty prevalence would be more evident in the older age ranges [51,54]. However, most of the studies included in this systematic review reported that the mean age of subjects was under 75 years old, and the influence of gender on frailty might not be noticeable. Therefore, consideration should be given to the influence of other factors on sex differences in further research. 

This is the first systematic review and meta-analysis on the prevalence of frailty among community-dwelling older adults in Asian areas. This study collected national representative population-based studies, which could compare frailty prevalence among countries. The elaborate search was conducted through multiple databases and reference lists of included studies to avoid missing significant evidence. The identified studies were analyzed with standardized processes and comprehensively assessed heterogeneity, methodological quality, and publication bias. Subgroup analyses based on the definition of frailty, geographical distribution, gender, age, marital status, living arrangement, smoking status, and alcohol drinking produced more insight into possible causes of heterogeneity between studies. 

Several limitations should be mentioned in our present study. Although we have collected the representative data for each country or region, there was a wide variety of research designs, sample sizes, sampling approaches, and the response rate that might affect the comparisons among included studies [14]. Therefore, the sources of heterogeneity have not been completely figured out with the evidence of a high degree of heterogeneity (the Higgin’s I^2^ value approaching 100%). Second, the number of studies available for each subgroup was less than ten, leading to a lack of statistical power in moderation analysis [61]. Third, no studies from Northern Asia and Central Asia might hinder the generalizability for all regions of Asia, suggesting the need for frailty research in these countries. Finally, the subgroups analyses did not imply any causal relationship variables [24]. Thus, it is required to recruit more studies from longitudinal follow-up studies and use a meta-regression technique to identify predictors of frailty.

Future research should investigate other associated risk factors (psychological and social aspects, country income level, etc.) by conducting additional stratified analyses to alleviate the degree of heterogeneity. Second, the gold standard for assessing frailty in older adults needs to be established to allow robust comparisons across populations. Finally, studies with the oldest population (80’s and 90’s) should be made without setting limitations for primary care and outpatient clinics. Further studies should also investigate frailty in young and middle-aged adults [62] to expedite the design and implementation of targeted treatments to prevent or alleviate the progression of frailty in community and clinical settings.

## 5. Conclusions

The prevalence of frailty in Asia is higher than the average global rate. One in every five community-dwelling older adults is frail in Asia. The prevalence of frailty was lower when the Fried phenotype (14.6%) was used but higher in the broad phenotype. Increasing age and being single was associated with a higher risk of frailty. The findings of this study provide a better understanding of the burden of frailty that occurs in Asian areas with rapidly aging populations. The differences in the prevalence of frailty and socioeconomic disparities among countries require health and social planning to be more detailed and appropriately focused on the actual needs of older subjects. Given the high prevalence, older people should be screened for frailty using acceptable instruments. This study encourages researchers to further investigate frailty and its aspects as a part of the public health interest to understand the underlying causes of frailty in this population. Clinicians and policymakers need to design appropriate interventions to prevent the progression of frailty and minimize its negative impact on health.

## Figures and Tables

**Table 1:** Characteristics of the studies included in the meta-analysis of prevalence of frailty.

First Author and Year of Publication	Country/Territory	United Nations Subdivisions	Effective Sample Size ^a^	Age (Mean or Median and Range)	% Female	Frailty Assessment Methods	Data Stratified According to Age	Data Stratified According to Sex	Prevalence of Frailty	Study Quality Assessment Score
Wu et al., 2017 [31]	China	Eastern Asia	5301	60+ (69.2 ± 7.0)	49.4	* Fried phenotype	yes	yes	7.0	8
Liu et al., 2018 [32]	China	Eastern Asia	11,165	65+ (82.6 ± 9.6)	52.0	Frailty Index–44 items	no	no	31.9	8
Yu et al., 2017 (Hong Kong) [30]	Hong Kong	Eastern Asia	4000	65+ (75.21)	50.0	Frailty Index–30 items	yes	yes	16.6	8
Yu et al., 2017 (Urban Taiwan) [30]	Taiwan	Eastern Asia	959	60+ (75.36)	45.78	Frailty Index–30 items	yes	yes	33.1	8
Yu et al., 2017 (Rural Taiwan) [30]	Taiwan	Eastern Asia	1412	60+ (75.45)	47.73	Frailty Index–30 items	yes	yes	38.1	9
Kendhapedi et al., 2019 (Southern India) [43]	India	Southern Asia	408	60+ (67.5 ± 6.62)	56.86	* Fried phenotype	no	yes	27.6	8
						Frailty index–40 items			59.2	
						Tilburg Frailty Indicator			62.6	
Kashikar & Nagarkar, 2016 (Western India) [44]	India	Southern Asia	250	65+ (73.9 ± 6.4)	50.0	* Fried phenotype	yes	yes	26.0	8
Pengpid et al., 2019 [35]	Indonesia	South-Eastern Asia	2630	60–101 (66.0)	50.60	* Fried phenotype	yes	yes	8.14	8
Murayama et al., 2020 [33]	Japan	Eastern Asia	2206	65+	56.4	* Fried phenotype	yes	yes	8.7	9
Boulos et al., 2016 [48]	Lebanon	Western Asia	1120	65+ (75.7 ± 7.1)	50.45	Study of Osteoporotic Fractures (SOF) index	yes	yes	36.4	8
Ahmad et al., 2018 (Rural Malaysia) [36]	Malaysia	South-Eastern Asia	2324	60+ (70.6)	61.8	* Fried phenotype	yes	yes	9.4	8
Norazman et al., 2020 (Urban Malaysia) [37]	Malaysia	South-Eastern Asia	301	60+ (67.08 ± 5.536)	69.4	* Fried phenotype	yes	yes	15.9	8
Devkota et al., 2017 [45]	Nepal	Southern Asia	253	60+	68.0	^b^ The Clinical Frailty Scale	no	yes	46.2	5
Vaingankar et al., 2017 [42]	Singapore	South-Eastern Asia	2102	60+ (69)	53.95	* Fried phenotype	yes	yes	5.7	8
Kang et al., 2017 [34]	South Korea	Eastern Asia	4352	65+ (72.6 ± 5.4)	57.4	Frailty index–42 items	no	yes	44.2	8
Siriwardhana et al., 2019 [46]	Sri Lanka	Southern Asia	746	60–94 (68)	56.7	* Fried phenotype	yes	yes	15.2	8
Srinonprasert et al., 2018 [40]	Thailand	South-Eastern Asia	8195	60+ (69.2 ± 6.8)	0.508	Frailty Index–30 items	yes	yes	22.1	7
Thinuan et al., 2020 (Northern Thailand) [38]	Thailand	South-Eastern Asia	1806	60–93 (70.74 ± 7.5)	70.54	* Fried phenotype	yes	yes	13.9	9
Chittrakul et al., 2020 (Chiang Mai Province) [39]	Thailand	South-Eastern Asia	367	65+ (73.22 ± 7.00)	64.6	* Fried phenotype	no	yes	8.7	8
Akin et al., 2015 [49]	Turkey	Western Asia	848	60+ (71.5 ± 5.6)	50.4	‡ Fried phenotype	yes	yes	27.8	9
						FRAIL scale			10.0	
Nguyen et al., 2019 [41]	Vietnam	South-Eastern Asia	512	60+	69.9	* Fried phenotype	yes	yes	21.7	8
Alqahtani et al., 2021 [50]	Saudi Arabia	Western Asia	486	71 (60–89)	34.7	* Fried phenotype	yes	yes	21.4	8
Delbari et al., 2021 [47]	Iran	Southern Asia	540	60+ (72.61 ± 8.72)	55.93	Frailty index–30 items	yes	yes	14.3	8

^a^ Where available, sample size includes those who died but excludes people lost to follow-up. * Fried phenotype with five criteria-weakness and slowness assessed using objective tests. ‡ Fried phenotype with four criteria: slowness, weakness, inactivity, and exhaustion. ^b^ The Frailty Scale from The Canadian Study of Health and Aging tool.

**Table 2:** Factors associated with pooled prevalence using Fried frailty phenotype.

Factors	Number of Datasets	Number of Frail Participants	Prevalence (%) (95% CI)	I^2^ (%)	*p*-Value for Difference
Region					
Eastern Asia	2	563	7.8 (3.9–13.0)	84	<0.01 **
South-Eastern Asia	7	996	11.3 (8.5–14.5)	96	
Southern Asia	3	292	22.5 (16.5–29.0)	93	
Western Asia	2	340	24.6 (17.4–32.7)	85	
Gender					0.67
Male	14	895	13.3 (10.1–16.9)	95.1	
Female	14	1348	15.6 (12.3–19.2)	95.7	
Age groups					0.01 *
60–64	11	511	8.1 (5.3–11.4)	95.8	
65–69	12	560	8.8 (6.1–12.0)	95.5	
70–74	12	718	14.4 (10.1–19.3)	96.1	
75–79	12	728	19.2 (14.2–24.7)	95.0	
80–84	12	641	29.8 (22.6–37.6)	93.7	
85+	12	630	34.0 (29.7–38.3)	73.2	
Marital status					0.02 *
Married	10	881	9.0 (7.1–10.9)	91.6	
Single	10	1211	21.5 (14.3–29.5)	98.1	
Living arrangement					0.18
Living alone	6	166	18.8 (11.8–27.0)	88.2	
Living with family	5	721	11.5 (8.3–15.1)	95.1	
Smoking status					0.81
No smoking	3	432	12.5 (7.0–19.2)	96.98	
Current smoking	4	138	11.3 (4.8–20.1)	92.57	
Alcohol Drinking					0.24
No drinking	2	243	16.4 (7.2–28.5)	96.4	
Current drinking	2	59	10.3 (1.0–27.6)	94.4	

Chi-square tests; ** *p* ≤ 0.01, * *p* ≤ 0.05.

## Data Availability

Data sharing is not applicable to this article.

## Supplementary Materials

The following supporting information can be downloaded at: https://www.mdpi.com/article/10.3390/healthcare10050895/s1, Figure S1: Map showing the geographical distribution of all included datasets (n = 23); Table S1: The Search Strategy; Table S2: Study Quality Assessment (Y—Yes, N—No, U—Unclear); Table S3: Characteristics of the studies included in the meta-analysis; Table S4: Estimates from Leave-One-Out Sensitivity Analyses for the prevalence of frailty.
