# Supplementary material for: Prevalence of Frailty among Community-Dwelling Older Adults in Asian Countries: A Systematic Review and Meta-Analysis
Source: Healthcare (Basel). 2022 May 12;10(5):895. doi: 10.3390/healthcare10050895 (PMC9140771; doi:10.3390/healthcare10050895)
Supplement: Supplementary file 1 [file healthcare-10-00895-s001.zip › healthcare-1670915-supplementary.pdf]

Supplementary Files

Table S1. The Search Strategy
Table S2. Study Quality Assessment (Y – Yes, N – No, U – Unclear)
Figure S1. Map showing the geographical distribution of all included datasets (n = 23)
Table S3. Characteristics of the studies included in the meta-analysis
Table S4. Estimates from Leave-One-Out Sensitivity Analyses for the prevalence of frailty

Table S1. The Search Strategy (01 January 2010 to 31 December 2020) (updated April 29, 2022)

Database	Search Strategy
Embase	'#1 'incidence'/exp OR incidence<br>#2 (prevalence:ti,ab,kw OR incidence:ti,ab,kw OR epidemiology:ti,ab,kw) AND 'frail elderly':ti,ab,kw
Cochrance	("frailty"):ti,ab,kw AND ("prevalence"):ti,ab,kw"
CINAHL	MW (prevalence or incidence or epidemiology or frequency or occurrence) AND MW (frailty or frail elderly or vulnerable elderly or vulnerability or functionally impaired elderly)
Ovid Medline	1. Frail Elderly.sh,kf.<br>2. (frail* or geriatric syndrome* or geriatric disorder*).ti,ab.<br>3. ((elder* or old* or senior* or geriatric*) adj4 function* adj4 (declin* or impair*)).af.<br>4. 1 or 2 or 3<br>5. Asian Countries.sh,kf.<br>6. (Eastern Asia* or Southern Asia* or South-Eastern Asia* or Western Asia* or Central Asia*).hw,kf,ti,ab,cp.<br>7. (Eastern adj3 Asia adj3 countr*).ti,ab.<br>8. (Southern adj3 Asia adj3 countr*).ti,ab.<br>9. (South-Eastern adj3 Asia adj3 countr*).ti,ab.<br>10. (Western adj3 Asia adj3 countr*).ti,ab.<br>11. (Central adj3 Asia adj3 countr*).ti,ab.<br>12. (Mongolia* or North Korea* or South Korea* or Japan* or China or Chinese or Maldives or Bhutan* or Sri Lanka* or Nepal* or Afghanistan or Iran* or Bangladesh* or Pakistan or India* or Brunei* or East Timor or East Timur or Timor Leste or Singapore* or Lao PDR or Laos or Cambodia* or Malaysia* or Malaya or Malay or Myanmar or Myanma or Thailand or Thai or Vietnam* or Viet Nam* or Philippines or Philipines or Phillippines or Phillipines or Cyprus or Bahrain or Qatar or Armenia* or Georgia or Georgian or Kuwait or Palestine or Oman or Lebanon or Israel or United Arab Emirates or Azerbaijan or Jordan* or Syria or Yemen* or Saudi Arabia or Iraq* or Turkey or Turkmenistan or Kyrgyzstan* or Tajikistan or Kazakhstan or Uzbekistan).hw,kf,ti,ab,cp.<br>13. 5 or 6 or 7 or 8 or 9 or 10 or 11 or 12<br>14. Prevalence/ or prevalence.mp.<br>15. incidence.mp. or Incidence/<br>16. epidemiology.mp. or Epidemiology/<br>17. 14 or 15 or 16<br>18. 4 and 13 and 17<br>19. limit 18 to English language

Table S2. Study Quality Assessment (Y – Yes, N – No, U – Unclear)

Author	Was the sample frame appropriate to address the target population?	Were study participants sampled in an appropriate way?	Was the sample size adequate?	Were the study subjects and the setting described in detail?	Was the data analysis conducted with sufficient coverage of the identified sample?	Were valid methods used for the identification of the condition?	Was the condition measured in a standard, reliable way for all participants?	Was there appropriate statistical analysis?	Was the response rate adequate, and if not, was the low response rate managed appropriately?	Total number of "Yes"
Wu et al.	Y	Y	Y	Y	U	Y	Y	Y	Y	8
Liu et al.	Y	Y	Y	Y	N	Y	Y	Y	Y	8
Yu et al. (Hong Kong)	Y	N	Y	Y	Y	Y	Y	Y	Y	8
Yu et al. (Urban Taiwan)	Y	N	Y	Y	Y	Y	Y	Y	Y	8
Yu et al. (Rural Taiwan)	Y	Y	Y	Y	Y	Y	Y	Y	Y	9
Kendhapedi et al. (Southern India)	Y	Y	Y	Y	N	Y	Y	Y	Y	8
Kashikar & Nagarkar (Western India)	Y	Y	Y	Y	N	Y	Y	Y	Y	8
Pengpid et al.	Y	Y	Y	Y	N	Y	Y	Y	Y	8
Murayama et al.	Y	Y	Y	Y	Y	Y	Y	Y	Y	9
Boulos et al.	Y	Y	Y	Y	N	Y	Y	Y	Y	8
Ahmad et al. (Rural Malaysia)	Y	Y	Y	Y	N	Y	Y	Y	Y	8
Norazman et al. (Urban Malaysia)	Y	Y	Y	Y	N	Y	Y	Y	Y	8
Devkota et al.	Y	N	Y	Y	N	Y	Y	Y	N	5
Vaingankar et al.	Y	Y	Y	Y	Y	Y	Y	Y	N	8
Kang et al.	Y	Y	Y	Y	N	Y	Y	Y	Y	8
Siriwardhana et al.	Y	Y	Y	Y	N	Y	Y	Y	Y	8
Srinonprasert et al.	Y	Y	Y	Y	N	Y	Y	Y	N	7
Thinuan et al. (Northern Thailand)	Y	Y	Y	Y	Y	Y	Y	Y	Y	9
Chittrakul et al. (Chiang Mai Province)	Y	Y	Y	Y	N	Y	Y	Y	Y	8
Akin et al.	Y	Y	Y	Y	Y	Y	Y	Y	Y	9
Nguyen et al.	Y	Y	Y	Y	N	Y	Y	Y	Y	8
Alqahtani et al	Y	Y	Y	Y	N	Y	Y	Y	Y	8
Delbari et al.	Y	Y	Y	Y	N	Y	Y	Y	Y	8

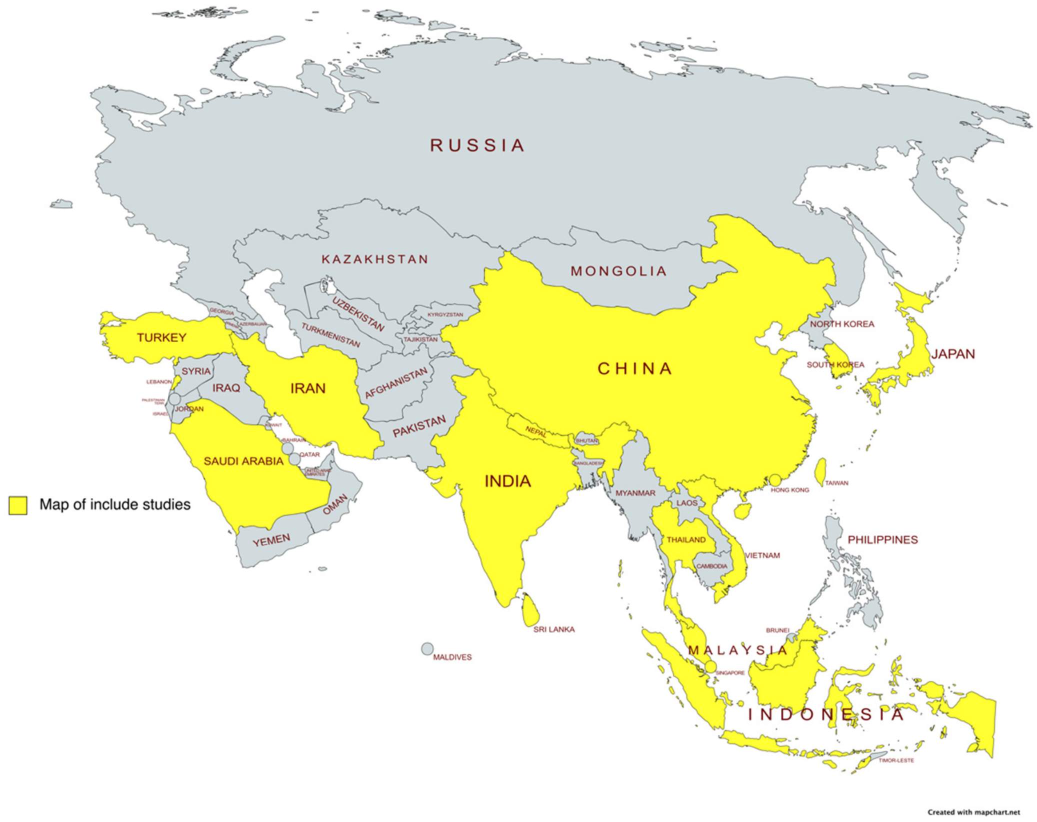

Figure S1. Map showing the geographical distribution of all included datasets (n = 23). Note. Countries that were included in the study are shown in yellow

Table S3. Characteristics of the studies included in the meta-analysis

First author and year of publication	Country	National Income Levels	Study Group/study setting/time period	Study de-sign	Sampling techinique	Frailty assessment methods	Prevalence (%), 95%CI		Risk factors assessed	Study strengths reported by authors	Study limitations reported by authors
							frailty	pre frailty			
Wu et al., 2017	China	Upper middle income	The China Health and Retirement Longitudinal Study 28 provinces in China (2011-2012)	Cross-sectional analysis	Multistage probability sampling	*Fried phenotype	7.0 (5.9–8.1)	51.2 (49.3-53.3)	Age, gender, education level, living areas, comorbidities, falls, disability, and functional limitation	- Using a widely validated frailty scale with cut-points allows screen for frailty in clinical practice.<br>- Examining regional disparities in frailty prevalence.<br>- Investigating the association of frailty and its biomarkers.	- The frailty prevalence was underestimated in the whole population.<br>- Frailty components were only measured once.<br>- Causal relationships were unable to establish between frailty status and medical conditions and disability.
Liu et al., 2018	China	Upper middle income	The Chinese Longitudinal Healthy Longevity Survey (CLHLS), 2002, 2005, and 2008 waves of in 22 provinces in China	A population-based cohort study	Non probability sampling	Frailty Index–44 items	31.9	47.9	Type of death	- Using a comprehensive frailty measurement in a large-scale cohort.<br>- The modification of the frailty measure demonstrated satisfactory validity.<br>- The sample size is the largest in prior studies on frailty transitions.	- The type of death consists of two domains; qualitative evaluation of the quality of death was unavailable.<br>- A higher percentage of attrition may have introduced bias.<br>- The short-term changes were not captured in this study using a period of three years.<br>- A deficit accumulation approach may overestimate frailty transitions.<br>- Using proxies to reduce nonresponse might introduce biases.
Yu et al., 2017 (Hong Kong)	Hong Kong	High income	Osteoporotic Fractures in Men (MrOs) and Women (MsOs) (2001–2003)	Cross-sectional analysis	Convenience sampling	Frailty Index–30 items	16.57	-	Age, gender, levels of physical activity, alcohol use, living alone.	- Using comparable measurements.<br>- Using performance measures of functioning for frailty.	- Using two different datasets with data collected at the different time points and the sampling strategies.

Yu et al., 2017 (Urban Taiwan)	Taiwan	High income	Taiwan Longitudinal Study on Aging (TLSA) (2003)	Cross-sectional analysis	A three-stage systematic random sampling method	Frailty Index–30 items	33.06	-			- The heterogeneity in frailty's definitions was used in different populations.
Yu et al., 2017 (Rural Taiwan)	Taiwan	High income				Frailty Index–30 items	38.1	-			
Kendhapedi et al., 2019 (Southern India)	India	Lower middle income	Four contiguous villages, in Southern India	Community based cross-sectional study	Census	*Fried phenotype	27.6 (18.9-28.1)	50.24	Age, gender, education level, socioeconomic status, and physical activity.	_	- Lack of any validated tool for frailty screening.<br>- Cognition was not measured.<br>- Self-reported frailty screening instruments might be an imprecision.<br>- Unable to establish a causal relationship between frailty and its factors.<br>- The sample size is not big enough to do regression analyses.<br>- The results may not be generalized to other settings.
						Frailty index–40 items	59.2	-			
						Tilburg Frailty Indicator	62.6	-			
Kashikar & Nagarkar, 2016 (Western India)	India	Lower middle income	Pune city, Maharashtra from December 2014 to January 2015	Cross-sectional study	Multistage random sampling	*Fried phenotype	26.0	63.6	Education level, disability, number of medicines per day, availability of support, fear of falling, not going out, falls, hospitalization, economic dependency, emotional support, and social isolation.	_	_
Pengpid et al., 2019	Indonesia	lower middle income	the Indonesia Family Life	Cross-sectional study	A multi-stage stratified	*Fried phenotype	8.14	61.6	Older age, marital status, country region, poor	_	- The nature of the cross-sectional research.

			Survey (IFLS-5) (2014–2015)		sampling design				cognitive functioning, loneliness, and functional disability.		- Excluding institutionalized elderly from surveys.<br>- The IPAQ is only used in populations 15–69 years old.<br>- The limitation of using single items in frailty definition, life satisfaction, and self-reported health status.
Murayama et al., 2020	Japan	High income	The National Survey of the Japanese Elderly (2012)	Cross-sectional analysis	A nationally representative survey	*Fried phenotype	8.7 (7.5–9.9)	40.8 (38.7 – 42.9)	Older groups, gender, and socioeconomic status.	_	- Using some modifiable components with the original version of the Fried criteria.<br>- A low response rate in the 2012 survey.
Boulos et al., 2016	Lebanon	Upper middle income	Rural Lebanese from March 2011 to 2012	A cross-sectional study	Multi staged cluster sampling	Study of Osteoporotic Fractures (SOF) index	36.4	30.4	Nutritional status, malnutrition, comorbidities, ADL independent, depressive symptoms, illiteracy, and cognitive status.	- A large sample size with a high response rate is potentially generalizable to the rural elderly population.<br>- The study is the first to evaluate the prevalence and associated factors.<br>- Using well-validated and widely used measures.	- Self-reporting data can be affected by memory and information bias caused by educational disparities.<br>- The inclusion of cognitively impaired individuals might result in underestimating the frailty status.<br>- Some private types of questions may be less reliable.<br>- Cultural adaptation is required in some items related to the MNA.
Ahmad et al., 2018 (Rural Malaysia)	Malaysia	Upper middle income	Kuala Pila, Negeri Sembilan, from November 2013 to	A prospective longitudinal study	A two-stage cluster sampling method	*Fried phenotype	9.4	57.9	Older age, gender, number of chronic diseases, cognitive function, socioeconomic	- The first study has identified various factors associated with frailty transition was conducted in rural communities.	- The recruitment of well-functioning participants excluded those with cognitive and motor impairment, leading to underestimated prevalence.

			February 2014 and a 12-month follow up from December 2014 to January 2015.						status, and physical activity.	- A large representative sample of a population.<br>- The objectively measured physical frailty in grip strength and walking speed domains to increase the accuracy of the results.<br>- Application of multiple imputations to dealing with missing data.	- A relatively short period of follow-up on the transition of frailty.<br>- Some modifiable components, such as weight loss, exhaustion, and low physical activity, may affect prevalence estimates of frailty.<br>- Some errors were observed in using a weight at 25 years of age to measure the weight loss.<br>- Lack of information about biological determinants, such as genetic factors, hormonal changes, and nutritional status.
Norazman et al., 2020 (Urban Malaysia)	Malaysia	Upper middle income		A cross-sectional study	The People Housing Project (Projek Perumahan Rakyat (PPR) at Kuala Lumpur between October 2018–January 2019.	*Fried phenotype	15.9%	72.8%	Age, gender, marital status, educational level, monthly household income (RM), presence of chronic diseases, functional status, cognitive status, depressive symptoms, BMI, mid-upper arm circumference, biochemical parameters.	-	- The observational study design is not appropriate to assess the causal relationship.<br>- Other confounding variables are still not unaccounted for this current study.<br>- The majority of participants aged 60–69 years old should not be generalized to all older adults. Thus, the association with predictors with advancing age could not be established.

Devkota et al., 2017	Nepal	Lower middle income	Health and Social Care Needs Assessment Survey of the Gurkha Welfare Pensioners in rural Nepal between March and April 2014.	A cross-sectional study	The convenience sampling method	[b] The Clinical Frailty Scale	46.2	-	Older age, smoking, living with son, breathing problems, pain and fatigue, dental health, and history of falls and fractures.	The study allows understanding the magnitude of comorbidity and frailty associated with aging in Nepal.	- Self-reporting of the medical conditions and their associated symptoms.<br>- The findings might not be necessarily generalizable to other similar populations in Nepal.<br>- There is potential for selection and sampling bias.
Vaingankar et al., 2017	Singapore	High income	the Well-being of the Singapore Elderly study (2013)	A cross-sectional single-phase survey	A disproportionate stratified sampling design	*Fried phenotype	5.7 (4.6–7.1)	40.1	Older age, social networks, having any care need, and a history of dementia, diabetes, cancer, respiratory problems or paralysis.	- This study used a nationally representative sample that is generalizable to the population.<br>- The study included additional information on psychosocial factors.<br>- Using a validated and widely screening tool.<br>- The inclusion of three major Asian ethnic groups: Chinese, Malay, and Indian.	- The cross-sectional studies cannot assess the cause and effect relationships.<br>- Were not established the validity and reliability of a self-report instrument for frailty.<br>- A low response rate and missing data on the frailty index.<br>- The use of self-report tools and lack of information about health and lifestyle behaviors.
Kang et al., 2017	South Korea	High income	the Korea National Health and Nutrition Examination Survey (2010 to 2012)	Cross-sectional study	Nationally representative samples	Frailty index–42 items	44.2	39.2	Hypertension (HTN)	- This study might be easily generalizable due to recruiting nationally representative samples.<br>- The results are statistical significance from multiple covariate adjustments.	- Unable to assess the causal and temporal relationship between frailty and HTN.<br>- Recall bias was reported in the study using self-reported questionnaires.<br>- Lack of information about questionnaires of the type of HTN medication and the

											number of concomitants used in KNHANES V.
Siriwardhana et al., 2019	Sri Lanka	Lower middle income	Rural areas of Kegalle district	A population-based cross-sectional study	A multistage probability sampling technique	*Fried phenotype	15.2 (12.3-18.6)	48.5	Age, longest-held occupation, education level	- This study was a large population-based study of a regional representative sample. - The rigorous methodology and measures were taken.<br>- Anthropometric measurements and physical fitness tests have shown excellent intra-rater reliability.<br>- The response rate was extremely high.<br>- Comparison of frailty distribution by income level and geographical regions in cross-country.	- The results are likely not generalizable.<br>- Frailty prevalence might be underestimated.<br>- Self-reports of physical activity and exhaustion might be influenced by recall bias.
Srinonprasert et al., 2018	Thailand	Upper middle income	The fourth Thai National Health Examination Survey (NHES-IV) in 2009	A cross-sectional survey	a nationally representative using a multi-stage, stratified sampling	Frailty index–30 items	22.1	-	Age, gender, good overall health status, smoking, wealth index quintile, BMI, gait speed, hand grip, history of falls, impaired BADLs, impaired IADLs, depressive mood, cognitive impairment, hypertension, diabetes, stroke, chronic obstructive pulmonary disease, death.	- The TFI followed a standardized procedure in a nationally representative, which has not been carried out in this region.<br>- TFI can predict longer-term mortality and efficiently be performed in community-dwelling older people	- Inadequate data about adverse outcomes to prevent falls, ADLs, and hospitalization.<br>- A self-reported functional status might not be reliable.

Thinuan et al., 2020 (Northern Thailand)	Thailand	Upper middle income	Northern Thailand from September 2017 to December 2018	A cross-sectional study	Stratified random sampling	*Fried phenotype	13.9	50.9	Age, education level, marital status, health perception, number of comorbidities, osteoarthritis and mid-arm circumference	_	- Some of Fried's criteria differ from previous studies making it challenging to compare results across studies.<br>- Evaluation of unexplained weight loss by self-reported data might be less accurate.<br>- Excluding mobility impaired, clinically unfit, and cognitively impaired individuals would not have reflected the actual prevalence.
Chittrakul et al., 2020 (Chiang Mai Province)	Thailand	Upper middle income	Saraphi District of Chiang Mai Province	A cross-sectional study	Stratified random sampling	*Fried phenotype	8.7	76.8	Sex, age, number of comorbidities, body mass index, fall risk	The first study asseses falls risk by separate aspects of the physiology	- The limitations of cross-sectional designs.<br>- The subgroup of frail individuals was too small.<br>- Future studies should consider a prospective cohort design study to measure the fall risk and other factors associated
Akin et al., 2015	Turkey	Upper middle income	The Kayseri Elderly Health Study (KEHES), urban area from August 2013 to December 2013.	A cross sectional population-based study	Stratified random sampling	‡Fried phenotype<br>FRAIL scale	27.8<br>10.0	34.8<br>45.6	Depressive mood, cognitive impairment, and malnutrition	_	- Physical activity was not assessed, leads to under-or overestimating the prevalence of frailty.<br>- The sample sizes of participants aged ≥85 years were relatively small.<br>- Cognitive assessment was assessed neither the FFI nor the FRAIL scale.
Nguyen et al., 2019	Vietnam	Lower middle income	Soc Son district, northern	A cross-sectional study	Random sampling	*Fried phenotype	21.7	65.6	Health-related quality of life (HRQOL).	_	- It is challenging to derive causal relationships from the cross-sectional analysis.

			Vietnam from February to April 2017								- This study utilized a self-reported questionnaire that may potentially lead to recall bias.<br>- Lack of data using the EQ-5D-5L to compare with other previous studies.<br>- It is not easy to generalize these findings to other settings in Vietnam.
Alqahtani et al., 2021	Saudi Arabia	High income	the Riyadh region in Alkharj city from August 2019 to June 2020	A cross-sectional study	Convenience sampling method	*Fried phenotype	21.4	47.3	Gender, age, marital status, living arrangements, education level, chronic conditions, BMI	_	- Lack of generalizability for all regions of Saudi Arabia.<br>- There is a need to control medications.<br>- The cross-sectional design is the limit causality relationship.<br>- Self-reported outcomes may potentially lead to less accuracy of the actual associations.<br>- Some components of frailty were evaluated using self-reported measures, which may lead to overestimation.<br>- Unable to control extraneous effects from psychology symptoms.
Delbari et al., 2021	Iran	Lower middle income	Five southwestern cities in Iran	A cross-sectional study	A proportional stratified sample	Frailty index	14.3	25.7	Age, gender, marital status, education level, living arrangement, economic status, job status,	The study helps determine high-risk groups.	- A cross-sectional study cann't establish a relationship between the outcome and predictor variables.<br>- The possible causes of failed convergence of regression parameters

resulted from defining the frailty status as a binary variable.<br>- The study results are likely applicable to other settings if not considering patients with sarcopenia and malignancy, which investigate by assessing frailty conditions.

Table S4. Estimates from Leave-One-Out Sensitivity Analyses for the prevalence of frailty

Study Omitted	Point estimate	Lower CI	Upper CI
Wu et al., 2017	0.21	0.16	0.27
Liu et al., 2018	0.20	0.15	0.26
Yu et al., 2017 (Hong Kong)	0.21	0.15	0.27
Yu et al., 2017 (Urban Taiwan)	0.20	0.15	0.26
Yu et al., 2017 (Rural Taiwan)	0.20	0.15	0.25
Kendhapedi et al., 2019 (Southern India)	0.20	0.15	0.26
Kashikar & Nagarkar, 2016 (Western India)	0.20	0.15	0.26
Pengpid et al., 2019	0.21	0.16	0.26
Murayama et al., 2020	0.21	0.16	0.27
Boulos et al., 2016	0.20	0.15	0.25
Ahmad et al., 2018 (Rural Malaysia)	0.21	0.16	0.27
Norazman et al., 2020 (Urban Malaysia)	0.21	0.16	0.26
Devkota et al., 2017	0.20	0.15	0.25
Vaingankar et al., 2017	0.21	0.16	0.27
Kang et al., 2017	0.20	0.15	0.25
Siriwardhana et al., 2019	0.21	0.16	0.26
Srinonprasert et al., 2018	0.20	0.15	0.27
Thinuan et al., 2020 (Northern Thailand)	0.21	0.16	0.27
Chittrakul et al., 2020 (Chiang Mai Province)	0.21	0.16	0.27
Akin et al., 2015	0.20	0.15	0.26
Nguyen et al., 2019	0.20	0.15	0.26
Alqahtani et al., 2021	0.20	0.15	0.26
Delbari et al., 2021	0.21	0.16	0.27
**Combined**	**0.21**	**0.16**	**0.26**
